# Reducing Strength Prevailing at Root Surface of Plants Promotes Reduction of Ag^+^ and Generation of Ag^0^/Ag_2_O Nanoparticles Exogenously in Aqueous Phase

**DOI:** 10.1371/journal.pone.0106715

**Published:** 2014-09-03

**Authors:** Peddisetty Pardha-Saradhi, Gupta Yamal, Tanuj Peddisetty, Peddisetty Sharmila, Shilpi Nagar, Jyoti Singh, Rajamani Nagarajan, Kottapalli S. Rao

**Affiliations:** 1 Department of Environmental Studies, University of Delhi, Delhi, India; 2 Department of Botany, University of Delhi, Delhi, India; 3 Department of Petroleum Engineering & Earth Sciences, University of Petroleum and Energy Studies, Dehradun, Uttarakhand, India; 4 Department of Chemistry, Indian Institute of Technology Delhi, Hauz Khas, New Delhi, India; 5 Department of Chemistry, University of Delhi, Delhi, India; RMIT University, Australia

## Abstract

Potential of root system of plants from wide range of families to effectively reduce membrane impermeable ferricyanide to ferrocyanide and blue coloured 2,6-dichlorophenol indophenol (DCPIP) to colourless DCPIPH_2_ both under non-sterile and sterile conditions, revealed prevalence of immense reducing strength at root surface. As generation of silver nanoparticles (NPs) from Ag^+^ involves reduction, present investigations were carried to evaluate if reducing strength prevailing at surface of root system can be exploited for reduction of Ag^+^ and exogenous generation of silver-NPs. Root system of intact plants of 16 species from 11 diverse families of angiosperms turned clear colorless AgNO_3_ solutions, turbid brown. Absorption spectra of these turbid brown solutions showed silver-NPs specific surface plasmon resonance peak. Transmission electron microscope coupled with energy dispersive X-ray confirmed the presence of distinct NPs in the range of 5–50 nm containing Ag. Selected area electron diffraction and powder X-ray diffraction patterns of the silver NPs showed Bragg reflections, characteristic of crystalline face-centered cubic structure of Ag^0^ and cubic structure of Ag_2_O. Root system of intact plants raised under sterile conditions also generated Ag^0^/Ag_2_O-NPs under strict sterile conditions in a manner similar to that recorded under non-sterile conditions. This revealed the inbuilt potential of root system to generate Ag^0^/Ag_2_O-NPs independent of any microorganism. Roots of intact plants reduced triphenyltetrazolium to triphenylformazon and impermeable ferricyanide to ferrocyanide, suggesting involvement of plasma membrane bound dehydrogenases in reduction of Ag^+^ and formation of Ag^0^/Ag_2_O-NPs. Root enzyme extract reduced triphenyltetrazolium to triphenylformazon and Ag^+^ to Ag^0^ in presence of NADH, clearly establishing potential of dehydrogenases to reduce Ag^+^ to Ag^0^, which generate Ag^0^/Ag_2_O-NPs. Findings presented in this manuscript put forth a novel, simple, economically viable and green protocol for synthesis of silver-NPs under ambient conditions in aqueous phase, using root system of intact plants.

## Introduction

Nanotechnology has witnessed spectacular advancement in fabrication and utilization of nanomaterials. Owing to small size and large surface to volume ratio, nanoparticles possess unique physicochemical and biological properties which differ entirely from the bulk material. Amongst different metal nanoparticles, the synthesis and application of silver NPs received wide attention, as silver nanoparticles find application in almost every sphere of life. Due to immense antimicrobial properties, silver NPs find wide application in medicine especially artificial teeth, bone coating, medical catheters, wound dressings besides surgical instruments. Silver nanoparticles are also extensively used in daily commodities such as cosmetics (viz. lotions, creams etc.), toothpastes, detergents, soaps, surface cleaners, room sprays, shoe insoles and textiles. Utility of silver nanoparticles in home appliances (such as washing machines, air and water filters), automotive upholstery, paints and food storage containers is also known [1]–[4]. Silver NPs find usefulness in sensing applications such as biolabeling, optical imaging of cancer and detection of DNA sequences [5], [6]. Electronic application of silver nanoparticles includes its usage for the preparation of optical devices, inks for circuit boards, high density recording devices, battery-based intercalation materials [6]–[9]. Large surface area of silver NPs provides high surface energy for catalysis [10]. Silver NPs of different sizes and shapes are routinely synthesized by various chemical and physical methods [11]–[13]. Various physical, chemical and physico-chemical approaches such as ion sputtering, laser ablation, inert gas condensation, mechanical milling, thermal or laser irradiation, chemical reduction, sol-gel technique, photochemical reduction and electrochemical techniques have so far been employed to generate metal NPs [12]–[14]. The Lee-Meisel method using sodium citrate along with heating for 1 h for generation of silver nanoparticles continues to be the standard by which other synthetic methods are compared. Although, the method is simple, however, it produces broad distribution of particle size in range of 20–600 nm [15], [16]. Recently, we demonstrated potential of biomolecules/components viz. yeast extract and mannitol to generate silver nanoparticles in size range of 10–50 and 10–20 nm, respectively, upon autoclaving with AgNO_3_ for 30 min at 121°C under a pressure of 1.06 kg/cm2. We had categorically reported that mannitol being a compatible solute (i.e. does not have any reported negative impact on living systems) can be exploited for large scale production of silver nanoparticles within a narrow range [11]. Although, this method reported by us is rapid, it required high temperature and pressure.

Owing to negative aspects of physical and chemical methods researchers across the globe have felt the need to develop alternate green/biological methods for generating NPs [17]–[19]. With an urge to develop environmentally benign green methods researchers looked at possible use of living systems, their components or dead biomass for synthesis of silver and other noble metal nanoparticles. Till date, researchers used (i) a variety of microorganisms, their cell free extracts and dead biomass (bacteria, actinomycetes and fungi) [11], [20]–[24]; (ii) cell organelles like chloroplasts [25]; (iii) extract from various components (viz. leaves, seeds, flowers, fruit or fruit peel, latex, tuber, bark etc.) of numerous plant species [17], [24]–[28] and (iii) live plants (which generate nanoparticles within their cells) to generate metal nanoparticles [26]–[32]. However, these biological methods also have certain limitations, such as (i) use of microorganism requires special facilities for their maintenance and safety measures [32]; (ii) it is difficult to precisely identify the biomolecule(s) amongst the cocktail of molecules in extracts of various biological materials/components, that are responsible for generation of silver NPs; (iii) it would be difficult to extract NPs synthesized intracellular in cells of plants or microorganisms and moreover, due to the presence of wide variety of biomolecules (having reducing strength) within the cells, NPs formed intracellular would be of broad size/shape range (31–32). In search for an alternate andideal green method, we felt it wise to exploit root system of intact plants for generating silver nanoparticles, as plant biologists clearly established that root system of plants possess immense reducing strength [32]–[36] and generation of metal nanoparticles from ions primarily involves reduction [10], [11], [31], [32], [36]. Therefore, the present investigations were carried with the aim to evaluate the potential of plants to generate silver nanoparticles exogenously at the root surface. To the best of our knowledge, no research team so far has made any efforts to evaluate if plant species possess potential to exogenously generate Ag NPs at the root surface. In this communication, using 16 plant species from 11 diverse taxonomic groups of angiosperms we are reporting for the first time that the roots system of intact plants can be exploited for exogenous generation of silver nanoparticles. We have also elaborated on the advantages of using root system of intact plants for rapid bulk synthesis of silver nanoparticles.

## Materials and Methods

### Plant material

Sixteen plant species from 11 distinct families of diverse taxonomic groups of Angiosperms were collected from wild and maintained garden beds of North/Main campus of University of Delhi, details of which are given in Table 1. In all the cases utmost care was taken to cause least damage to the plants as well as to the root system at the time of collection.

**Table 1:** Details of plant species used for exogenous generation of silver NPs by root system of intact plants.

Botanical Name	Common Name	Family	Size of NPs (nm)
*Amaranthus gracilis*	Slender Amaranth	Amaranthaceae	10–30
*Cannabis sativa*	Hemp	Cannabinaceae	20–50
*Catharanthus roseous*	Madagascar Periwinkle	Apocynaceae	10–30
*Cynodon dactylon*	Couch Grass	Poaceae	20–50
*Euphorbia hirta*	Asthma Plant	Euphorbiaceae	20–40
*Medicago sativa*	Alfalfa	Fabaceae	10–40
*Ocimum sanctum*	Holy Basil	Lamiaceae	20–50
*Phyllanthus fraternus*	Gulf Leaf Flower	Euphorbiaceae	10–50
*Portulaca grandiflora*	Moss Rose	Portulacaceae	20–50
*Tagetes erecta*	Marigold	Asteraceae	5–30
*Vernonia cinerea*	Little Ironweed	Asteraceae	20–50
*Brassica juncea*	Indian Mustard	Brassicaceae	5–40
*Cicer arietinum*	Chickpea	Fabaceae	5–30
*Lycopersicon esculentum*	Tomato	Solanaceae	10–40
*Triticum aestivum*	Wheat	Poaceae	10–30
*Vigna mungo*	Black Gram	Fabaceae	10–30

### Raising plants under sterile conditions

Seeds of *Brassica juncea* cv. Varuna, *Vigna mungo* cv. PS-1, and *Lycopersicon esculentum* cv. Pusa Ruby were purchased from Seed Sales Counter, Indian Agricultural Research Institute (IARI), New Delhi, and that of *Triticum aestivum* cv. H1-1544 and *Cicer arietinum* cv. PG-114, were gifted by Dr. A.N. Mishra (Head, IARI Regional Station, Indore, India) and Dr. Jitendra Kumar (Principal Scientist, Pulse Division, IARI, New Delhi, India), respectively. Seeds were treated with 0.5% cetrimide for 5 min, washed with distilled water; surface sterilized with 0.1% (w/v) mercuric chloride for 3 min and washed thoroughly with sterile distilled water. Capped glass bottles of (2.5×5″) dimensions containing glass beads (∼75 g) with 25 ml of mineral growth medium [consisting of 2500 mg L^−1^ KNO_3_, 250 mg L^−1^ MgSO_4_, 150 mg L^−1^ CaCl_2_, 150 mg L^−1^ NaH_2_PO_4_, 134 mg L^−1^ (NH_4_)_2_SO_4_, 10 mg L^−1^ MnSO_4_, 3 mg L^−1^ H_3_BO_3_, 2 mg L^−1^ ZnSO_4_, 0.75 mg L^−1^ KI, 0.25 mg L^−1^ Na_2_MoO_4_ and iron source (27.8 mg L^−1^ FeSO_4_ and 37.3 mg L^−1^ EDTA)] [37] were sterilized by autoclaving at a pressure of 1.06 Kg cm^−2^ and at temperature of 121°C for 30 min. Surface sterilized seeds were sown in these bottles under sterile conditions, in laminar air flow. For present investigations 20 day old plants of *L*. *esculentum*, 9–10 day old plants of *B. juncea, C. arietinum*, and 4–5 day old plants of *T. aestivum* and *V. mungo* were carefully removed from glass beads with least damage to the root system, and incubated for 6–12 h in AgNO_3_ solution.

### Determining root surface redox activity

In order to determine reduction potential associated with root surface, roots of intact plants were submerged in 0.1 mM DCPIP solutions. Reduction potential of roots of intact plants was determined by monitoring decrease in the optical density at 600 nm due to reduction of DCPIP to DCPIPH_2_. Capacity of roots of intact plants to reduce DCPIP was expressed in terms of nano moles of DCPIP reduced h^−1^g^−1^ root fresh weight. Reduction potential associated with roots of intact plants of different plant species was also determined using ferricyanide which is a membrane impermeable electron acceptor [32]–[35]. Potassium ferricyanide {K_3_[Fe(CN)_6_]} was used at 1 mM concentration for evaluating redox activity of roots. The reduction of ferricyanide was recorded by measuring decrease in the optical density at 420 nm. Capacity of roots of intact plants to reduce ferricyanide was expressed in terms of nmoles of ferricyanide reduced h^−1^g^−1^ root fresh weight.

All experiments were conducted atleast five independent times. The data expressed as mean ±SE have been statistically analyzed using ANOVA (Analysis of Variance) through SPSS (Statistical Package for Social Sciences) version 16.0. The differences between means were tested for significance by Duncan's multiple range test (DMRT) at p≤0.05.

### Incubation of plants with salt solutions

For evaluating the impact of Ag^+^, and potential of plants to generate silver NPs, plants were exposed to different concentrations (viz. 0.5, 1 and 2 mM) of silver nitrate (AgNO_3_) solutions, prepared in double distilled water. All the plants placed in various salt solutions were incubated at room temperature (25±2°C) with a 16/8 h light/dark cycle. White fluorescent light (Phillips, India) with a light intensity of 120 µmol m^−2^ s^−1^ photon flux density was used for illuminating the plants.

### Incubation of plants under strict sterile conditions with salt solutions

For evaluating potential of plants to generate Ag-NPs they were exposed to different concentrations (viz. 0.01, 0.05, 0.1, 0.25, 0.5, and 1 mM) of AgNO_3_ prepared in sterile double distilled water. Plants were incubated at room temperature (25±2°C) with a 16/8 h light/dark cycle with a light intensity of 120 µmol m^−2^ s^−1^ photon flux density. For experiments carried under strict sterile conditions the stock solutions of filter sterilized Ag was used. Different concentrations were prepared from this stock using sterile double distilled water in laminar air flow. The glassware(s) used in these experiments were sterilized by autoclaving at a pressure of 1.06 Kg cm^−2^ and at temperature of 121°C for 30 min. After washing roots 2–3 times with sterile double distilled water, the plants raised under sterile conditions were transferred to 25×200 mm sterile Borosil tubes with their roots immersed in sterile salt solutions, in laminar air flow. The exogenously generated silver nanoparticles were characterized through UV-Vis spectral studies, TEM investigations coupled with EDX and PXRD studies.

### UV-Vis spectral studies

UV-Vis absorption spectra of various test solutions in which roots of intact plants were submerged for different time intervals were recorded between 190–1100 nm using Specord 200 Analytikjena UV-Vis spectrophotometer. The formation of Ag nanoparticles was indicated by the appearance of characteristic absorption peak generally at 410–480 nm.

### Transmission electron microscopic (TEM) investigations

AgNO_3_ solutions incubated with roots of intact plants for duration of ∼24 h were collected and subjected to sonication for 30 min to minimize primary particle agglomeration. 10 µl of the resulting colloidal solution was drop coated onto a 200-mesh copper grid covered with an ultrathin continuous C film in a desiccator. The grids were viewed in transmission electron microscope of Technai G^2^ T30 U-TWIN at a voltage of 300 kV. Hardware associated with the machine also allowed (i) energy dispersive X-ray (EDX spectra), and (ii) Selected area electron diffraction (SAED) pattern measurements. EDX spectra indicated the composition of isolated nanoparticles and SAED patterns indicated the crystalline/amorphous nature of the nanoparticles that were fabricated and released by the plants into the solutions.

### Powder X-ray diffraction (PXRD) Studies

AgNO_3_ solutions incubated with roots of intact plants was drop coated on Silica surface and dried. The PXRD pattern was collected using Rigaku Rotaflex RAD-B or Bruker or Pan Analytika instruments using Cu target CuK(α) at the rate of 0.020 step in 1.2 s in the 2 theta range 30–80°.

### Detection of proteins, amino acids or phenolics in incubation medium

Roots of intact plants were incubated in sterile double distil water for a duration of 24 h. This incubation medium was then tested for the presence of proteins, phenolics and amino acids using standard protocols [38]–[40].

### Comparative study of roots of plants with sodium citrate

The efficiency of roots of plants was compared to one of the most widely used reductant viz. sodium citrate. The stock solution of AgNO_3_ was filter sterilized and various concentrations viz. 0.01, 0.05, 0.1, 0.25, 0.5 and 1 mM were prepared using sterile double distilled water.

### Determination of dehydrogenase activity

#### In vitro

Roots of (i) *Triticum aestivum* seedlings (4–5 d old) raised under sterile conditions; and (ii) *Portulaca grandiflora* plants raised in earthen pots were homogenized independently in 50 mM phosphate buffer (pH-7.2) containing 5 mM EDTA and 5% of polyvinylpyrrolidone and centrifuged at 16000 xg at 4°C. Supernatant was used as the crude enzyme extract for determining dehydrogenase activity following modified protocol of Yamauchi and co-workers [41]. The reaction mixture for determining the dehydrogenase activity consisting of 500 µl crude enzyme extract, 10 µmoles of 2,3,5-triphenyltetrazolium chloride and 2 µmoles of NADH in a final volume of 4 ml of 100 mM phosphate buffer (pH 7.6), was incubated at 37°C for 6 h. Subsequently, the reaction mixtures were centrifuged at 10000 xg for 20 min and the pellet was dissolved in 4 ml ethanol. The quantity of 1,3,5-triphenylformazan formed due to dehydrogenase activity was determined by taking absorbance at 484 nm. The dehydrogenase activity was finally expressed as nmoles of formazan formed g^−1^ fresh weight of root h^−1^.

Simultaneously, the potential of dehydrogenases present in crude enzyme extract to reduce Ag^+^ and form silver-NPs determined by incubating 500 µl of crude enzyme extract with 100 mM phosphate buffer (pH-7.6) containing 1 µmole AgNO_3_ and 2 µmoles NADH in a final volume of 4 ml at 37°C for 6 h. The presence of silver-NPs in resultant reaction mixture was confirmed by (i) absorption spectra which showed Ag-NPs specific peak; (ii) transmission electron microscope.

#### In vivo

Roots of intact plants, of (i) *Triticum aestivum* (4–5 d old) raised under sterile conditions; and (ii) *Portulaca grandiflora* raised in earthen pots, were incubated in phosphate buffer (pH- 7.6) containing either 2,3,5-triphenyltetrazolium chloride or 0.25 mM AgNO_3_. After incubating for different time intervals (6 to 24 h), (i) resultant incubation medium was centrifuged at 10000 xg for 20 min and the pellet consisting of 1,3,5-triphenylformazan was dissolved in 95% ethanol; and (ii) root system of plants were excised, cut in to segments and dipped in 95% ethanol for 12 h to extract out 1,3,5-triphenylformazan. The formazan formed due to dehydrogenase activity was determined by measuring absorbance at 484 nm. The dehydrogenase activity was finally expressed as nmoles of formazan formed g^−1^ fresh weight of root h^−1^.

## Results and Discussion

It is well established that generation of silver nanoparticles similar to other metal nanoparticles primarily involves reduction of their ions [10], [11]. Earlier, it had been unequivocally proven that root system of plant species possess strong reducing capacity [32]–[36].

**Figure 1:**
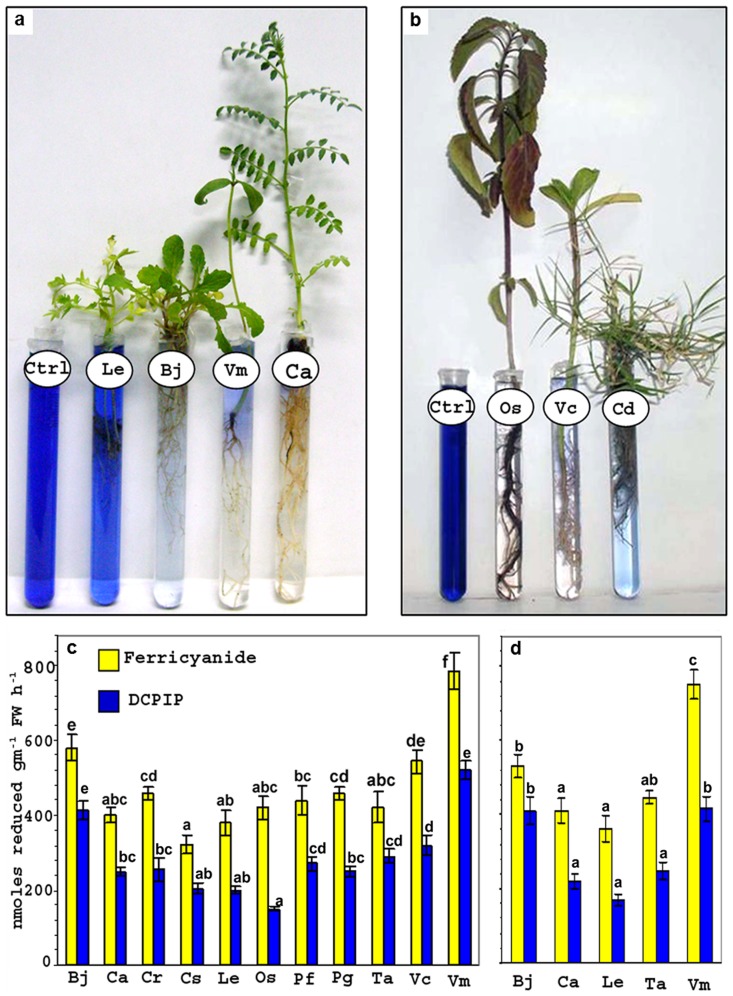
Potential of root system of plants to reduce DCPIP (0.1 mM) and ferricyanide (1 mM). Reduction of DCPIP (a–d) and ferricyanide (c–d) by root system of intact plants of *Lycopersicon esculentum* (Le), *Brassica juncea* (Bj), *Vigna mungo* (Vm), *Cicer arietinum* (Ca), *Ocimum sanctum* (Os), *Catharanthus roseous* (Cr), *Cynodon dactylon* (Cd), *Cannabis sativa* (Cs), *Phyllanthus fraternus* (Pf), *Portulaca grandiflora* (Pg), *Triticum aestivum* (Ta) and *Vernonia cinerea* (Vc) under non-sterile (a–c) and sterile (d) conditions. Ctrl represents 0.1 mM DCPIP. Vertical lines on bars represent mean ± standard error, n = 5. Values designated by different letters above bars are significantly different between plant species at p ≤ 0.05 level (Duncan's multiple range test).

During present investigations root system of intact plants of all 16 plant species, from 11 diverse families of angiosperms effectively reduced artificial electron acceptors (DCPIP) and ferricyanide within duration of 6-12 h. As is evident from Fig. 1 root system of plants reduced (i) blue colored DCPIP to colorless DCPIPH_2_ and (ii) ferricyanide to ferrocyanide. Earlier, Rubinstein and co-workers recorded reduction of membrane impermeable ferricyanide to ferrocyanide in the solutions in which roots of intact plants were incubated [34]–[35]. Our findings similar to the findings of Rubinstein *et al.* [34] clearly established that root surface could exogenously reduce ferricyanide to ferrocyanide. This prompted us to test if root system of intact plants can exogenously reduce Ag^+^ to Ag^0^ at the root surface and generate silver nanoparticles in aqueous phase under ambient conditions.

**Figure 2:**
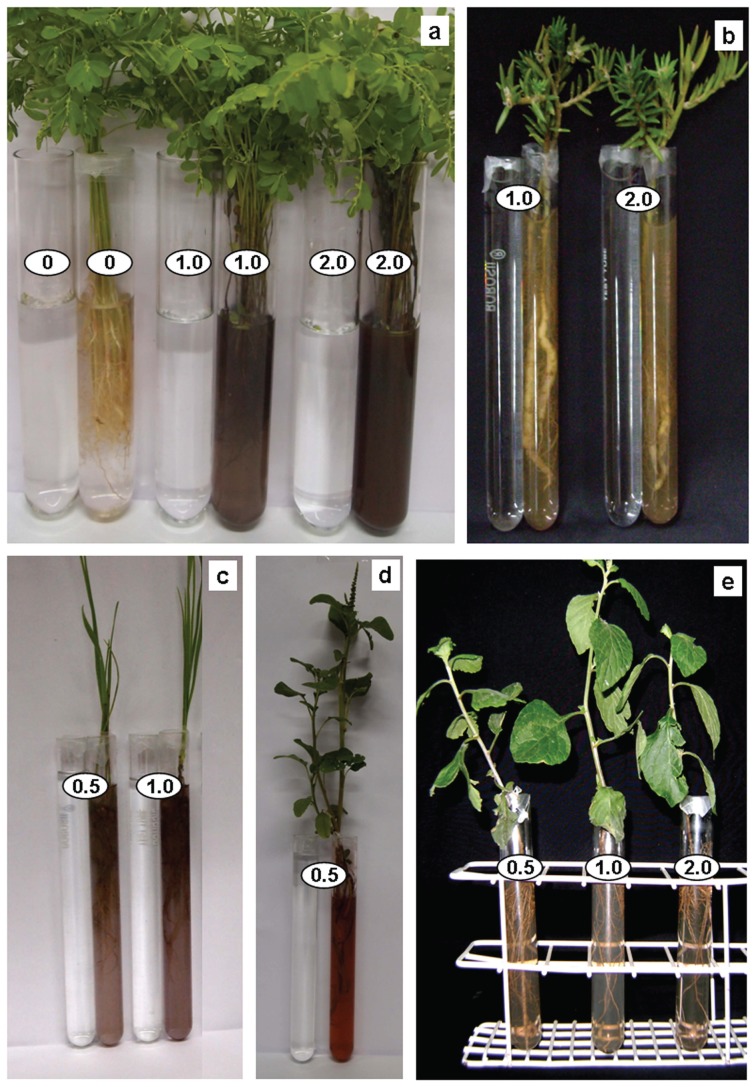
Potential of root system of intact plants to generate silver NPs. Root system of intact plants of (a) *Phyllanthus fraternus*, (b) *Portulaca grandiflora*, (c) *Triticum aestivum*, (d) *Amaranthus gracilis* and (e) *Vernonia cinerea* exhibitingpotential to alter clear colorless AgNO_3_ of different concentrations (mM) turbid brown. No color change noted in the tubes containing different concentrations of AgNO_3_ incubated under similar conditions without plants (a–d).

**Figure 3:**
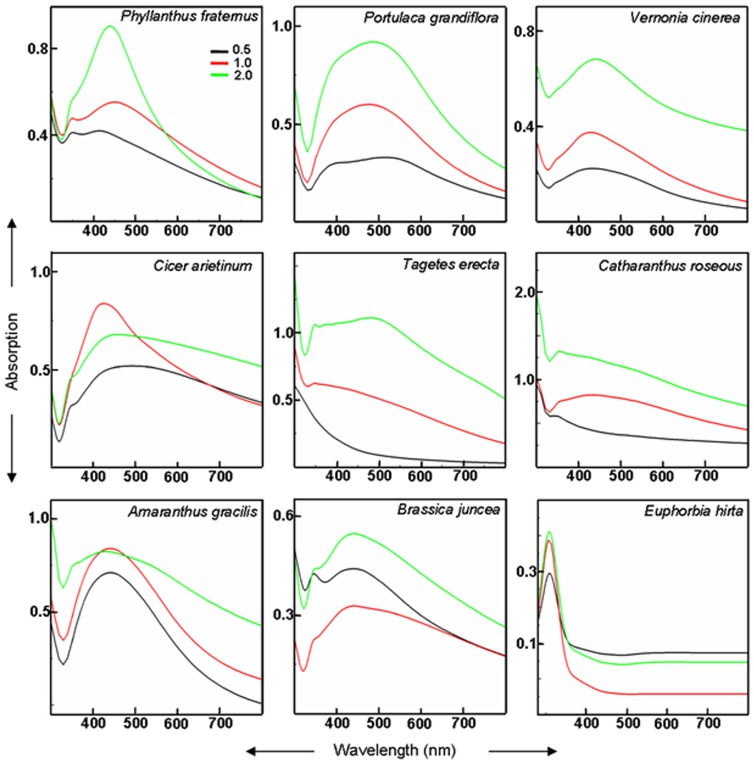
UV-Vis absorption spectra of 0.5, 1.0 and 2 mM AgNO_3_ exposed to root system of intact plants of different plant species for 12 h.

As anticipated, root system of intact plants of all plant species tested, altered color of clear AgNO_3_ solution, turbid brown, within a duration of 6–12 h (Fig. 2a–e), probably due to reduction of Ag^+^ and formation of silver nanoparticles. It is known that clear AgNO_3_ solutions turn brown upon generation of silver nanoparticles [11], [42]. In general, the UV-Vis absorption spectra of these brown colored colloidal solutions showed absorption maxima between 380 to 450 nm (Fig. 3). Absorption peak in this range has been reported to arise due to surface plasmon resonance in silver NPs [11]. The intensity and position of absorption peak varied depending on the concentration of Ag^+^ and plant species used (Fig. 3).

**Figure 4:**
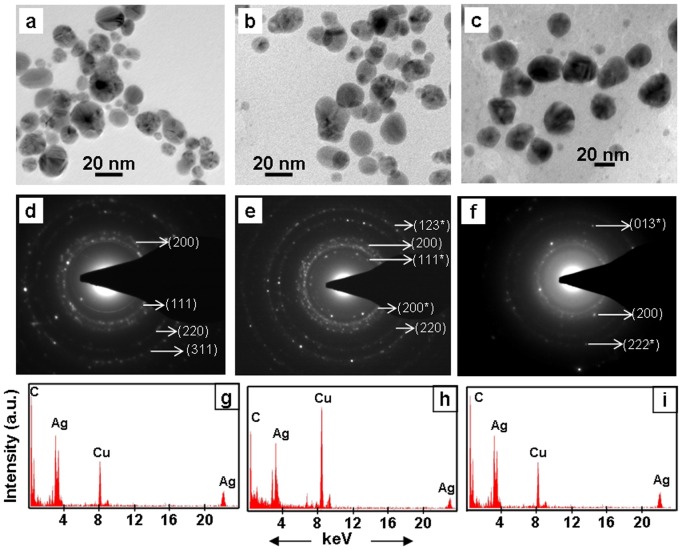
TEM images (a–c), SAED (d–f) and EDX (g–i) of silver NPs synthesized exogenously by root system of intact plants of *Tagetes erecta* (a, d, g), *Catharanthus roseous* (b, e, h), *Euphorbia hirta* (c, f, i). SAED show Bragg reflections characteristic of crystalline Ag^0^ () and Ag_2_O (*).

**Figure 5:**
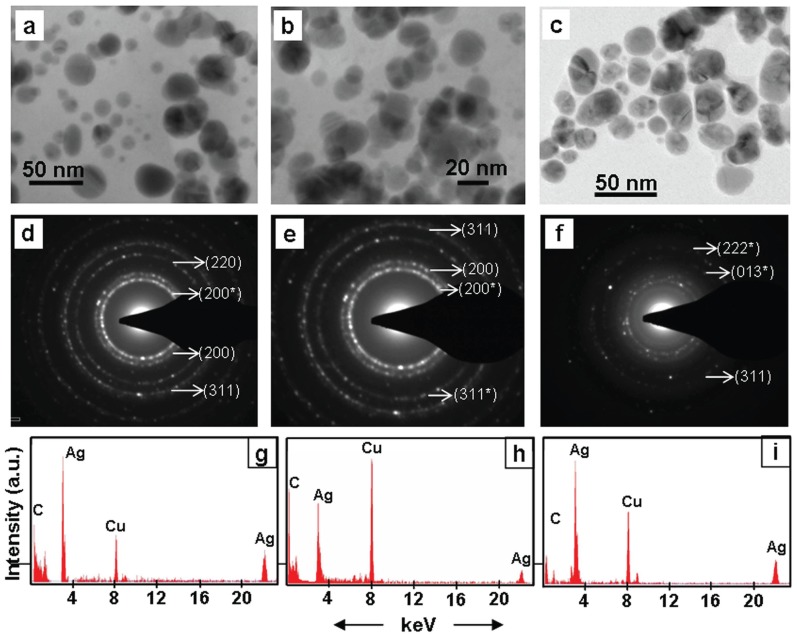
TEM (a–c), SAED (d–f) and EDX (g–i) of silver NPs synthesized exogenously by roots system of intact plants of *Brassica juncea* (a, d, g), *Cicer arietinum* (b, e, h), and *Phyllanthus fraternus* (c, f, i). SAED show Bragg reflections characteristic of crystalline Ag^0^ () and Ag_2_O (*).

**Figure 6:**
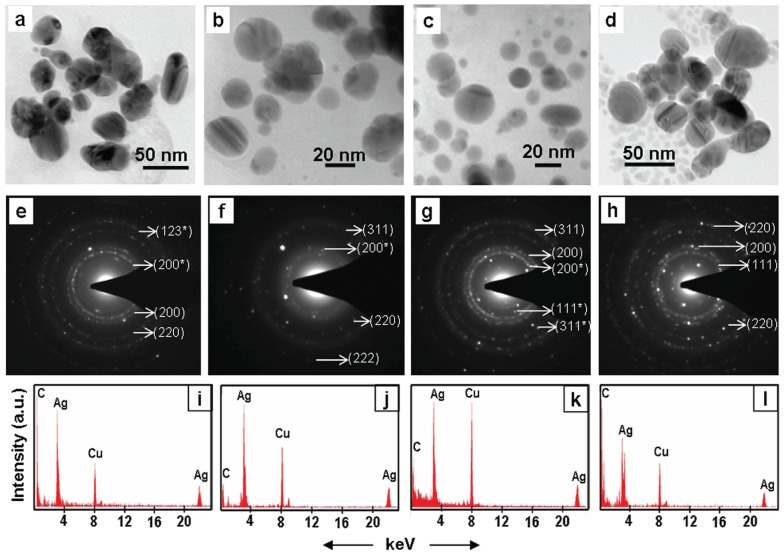
TEM (a–d), SAED (e–h) and EDX (i–l) of silver NPs synthesized exogenously by root system of intact plants of *Cynodon dactylon* (a, e, i), *Portulaca grandiflora* (b, f, j), *Lycopersicon esculentum* (c, g, k) and *Vernonia cinerea* (d, h, l). SAED show Bragg reflections characteristic of crystalline Ag^0^ () and Ag_2_O (*).

Transmission electron microscopic (TEM) investigations confirmed the presence of distinct NPs in brown colloidal solutions that resulted due to incubation of root system of intact plants in AgNO_3_ solutions (Figs. 4–6, Table 1). In general, the NPs were in the range of 5–50 nm and spherical (Figs. 4–6). These results are in accordance with the UV-Vis absorption spectra of the colored colloidal solutions that showed surface plasmon resonance (SPR) peak centered around 430 nm, implying the presence of spherical NPs [43]. Energy dispersive X-ray (EDX) studies confirmed the presence of Ag in these NPs (Figs. 4–6). The EDX pattern (Figs. 4–6) collected from these nanoparticles showed distinct peaks at 3.40 keV and 22 keV corresponding to Ag, while the peaks situated at binding energies of 8.06 and 1 keV correspond to Cu and C, respectively. The peaks of C and Cu arose due to their presence as an integral component of carbon coated copper grids. The presence of distinct rings corresponding to Bragg reflections in selected area electron diffraction (SAED) pattern established that these silver NPs were crystalline (Figs. 4–6).

**Figure 7:**
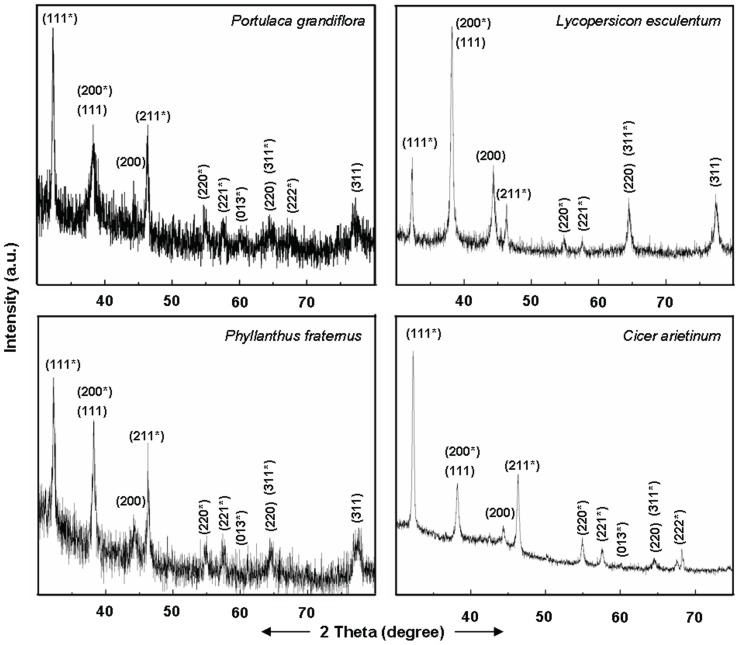
PXRD pattern of silver NPs. The PXRD pattern of silver NPs synthesized exogenously by roots system of intact plants of various plant species showing Bragg reflections characteristic of crystalline face-centred cubic structure of Ag^0^ () and cubic structure of Ag_2_O (*).

Powder X-ray diffraction (PXRD) patterns of silver nanoparticles generated exogenously by root surface of intact plants showed presence of distinct peaks, corroborating their crystalline nature. Irrespective of the plant species used, the PXRD patterns of silver nanoparticles formed exogenously by root of intact plants matched with the standard diffraction pattern of Joint Committee on Powder Diffraction Standards (JCPDS) No. 89–3722, confirming the face-centered cubic (fcc) structure of Ag^0^, and 76–1393 characteristic of Ag_2_O with cubic geometry (Fig. 7). These PXRD results revealed that the silver nanoparticles formed by the root surface of plant species are constituted of Ag^0^ and Ag_2_O. During earlier investigations also we recorded susceptibility of Ag^0^ NPs to oxidation and accordingly, recorded formation of a mixture of Ag^0^/Ag_2_O NPs by yeast extract, mannitol as well as yeast extract-mannitol medium [11]. Levard and co-workers [44] also reported the susceptibility of Ag^0^ NPs to oxidation under ambient conditions [44]. Therefore, we believe that some of the Ag^0^ NPs formed by reduction of Ag^+^ at the root surface were oxidized to Ag_2_O NPs in aqueous phase under ambient conditions. Recently, we also reported that the root surface of plants possesses potential to form iron oxy-hydroxide NPs [36].

### Possible mechanism of generation of silver nanoparticles at the root surface

Earlier, two plant species namely, *Medicago sativa* (alfalfa) and *Brassica juncea* (mustard) which are considered to be metallophytes have been reported to synthesize silver nanoparticles within their cells [29]–[31]. Gardea-Torresdey *et al.* [29] reported that alfalfa roots absorb Ag^0^ from agar medium and transfer it to shoot in the same oxidation state, where these silver atoms (Ag^0^) arrange themselves to form nanoparticles. However, these researchers neither addressed the mode of reduction of Ag^+^ to Ag^0^ in agar medium nor discussed about any factor (if any) responsible for arrangement of Ag^0^ to form nanoparticles in the shoots. Harris and Bali [30] noted formation of silver nanoparticles within the cells of plants of *M*. *sativa* and *B*. *juncea* exposed to AgNO_3_ solution, but these researchers also remained silent on mode of reduction of Ag^+^ and generation of Ag nanoparticles. Subsequently, Beattie & Haverkamp [31] reported synthesis of silver nanoparticles in cells of *B*. *juncea*, predominantly in chloroplast. The later researchers believed that the reducing sugars could play a role in reduction of Ag^+^ and generation of silver NPs. However, plant cells besides having reducing sugars also possess number of other biomolecules such as phenolics, organic acids, amino acids etc. which have been shown to be potent reductants for reduction of Ag^+^ to generate silver nanoparticles [22], [27], [45].

During present investigations, root system of all plant species tested possessed potential to exogenously reduce Ag^+^ and form silver nanoparticles when incubated with AgNO_3_ solution. Roots of plants are known to exude a variety of biomolecules viz. organic acids, amino acids, phenolics etc. [46] which have been shown to possess capacity to reduce Ag^+^ and generate silver nanoparticles [11], [47], [48]. To evaluate if capacity of root system of plants to reduce Ag^+^ and generate Ag nanoparticles exogenously, recorded during present investigations is due to biomolecules that are exuded by the roots, 2 sets of investigations were carried. In the first set of investigations, we tested for presence of phenolics, amino acids and proteins in the medium in which roots of intact plants were incubated for 24 h, but, did not find any detectable levels of these biomolecules. Subsequently, the incubation medium in which roots of intact plants were incubated for 24 h, was tested directly for its potential to generate silver nanoparticles. Neither color change nor presence of any silver nanoparticles was recorded. However, as stated above roots of intact plants incubated in AgNO_3_ solution could reduce Ag^+^ and generate silver nanoparticles within a duration of 6–12 h. Infact, earlier researchers also categorically demonstrated that the reduction of membrane impermeable ferricyanide to ferrocyanide is not due to any of the lechates from the roots [34], [35]. Therefore, our findings clearly indicated that the exogenous reduction of Ag^+^ and generation of silver nanoparticles by root surface of intact plants within a duration of 6–12 h is not due to biomolecules (if any) released by the root system of intact plants.

Another likely possibility for the exogenous generation of silver nanoparticles could be due root associated microorganisms, as microorganisms can generate silver nanoparticles [21], [22]. Infact, it has been reported that the root associated microorganisms are involved in the reduction of metal ions [49], [50]. In order to validate if microorganisms (if any) harbored by the root system in any way are essential for reduction of Ag^+^ and generation of silver nanoparticles exogenously, investigations were carried with *Brassica juncea*, *Triticum aestivum*, *Vigna mungo*, *Cicer arietinum* and *Lycopersicon esculentum* plants under strict sterile conditions.

**Figure 8:**
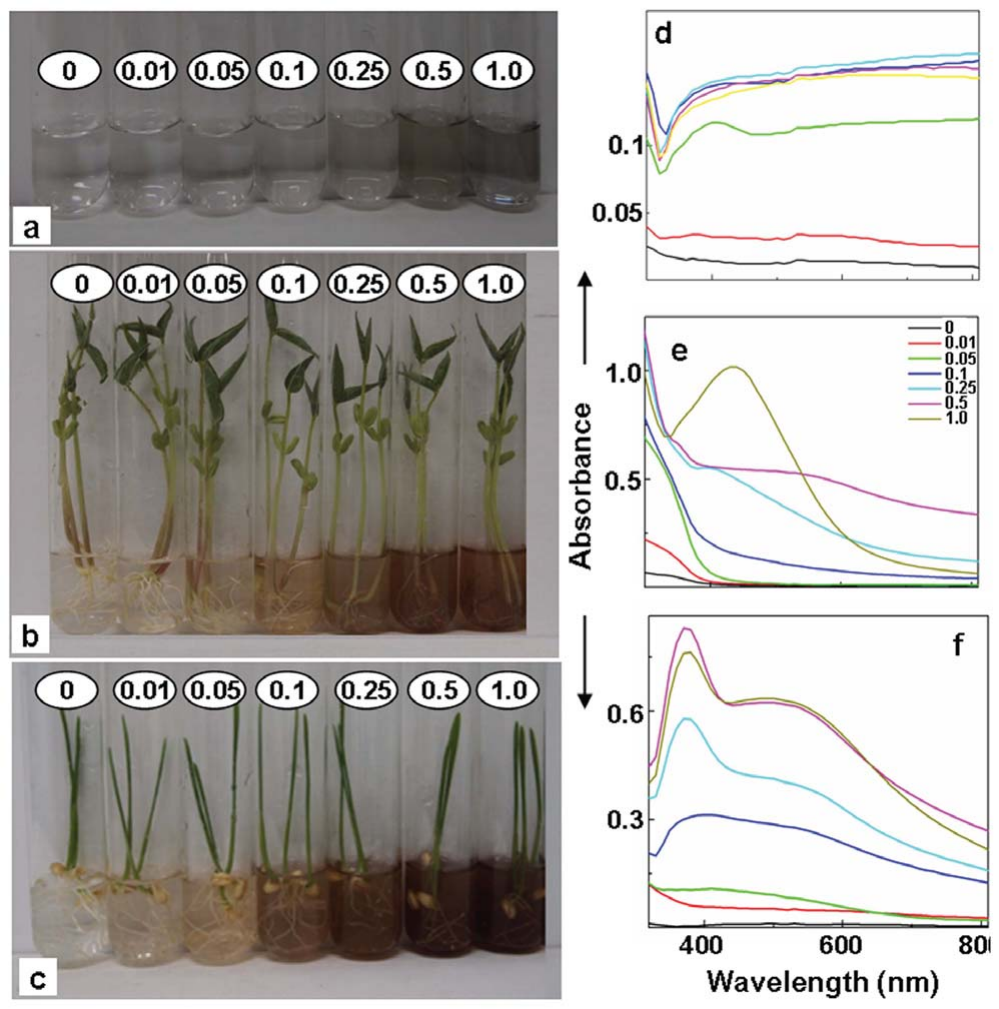
Potential of sodium citrate and root system of intact plants of 4 day old *Vigna mungo* and *Triticum aestivum* to generate silver NPs. 1% sodium citrate (a) and root system of intact plants of *V*. *mungo* (b) and *T*. *aestivum* (c) incubated in AgNO_3_ of different concentrations (mM) for 6 h, showing alteration in color and turning clear solution colloidal under sterile conditions at room temperature. UV-Vis spectra of resultant colloidal solutions formed by 1% sodium citrate (d), *V. mungo* (e) and *T. aestivum* (f).

**Figure 9:**
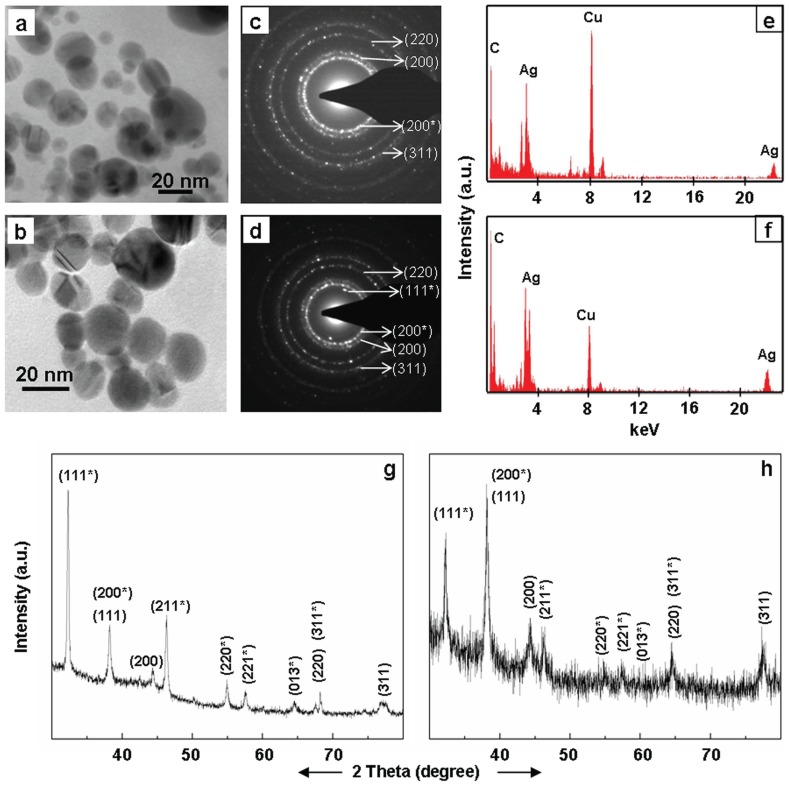
TEM images (a–b), SAED (c–d), EDX (e–f) and PXRD (g–h) of silver NPs synthesized exogenously by root system of intact plants of *Vigna mungo* (a, c, e, g) and *Triticum aestivum* (b, d, f, h). Bragg reflections characteristic of crystalline face-centred.

Root system of all the plant species raised and assessed under strict sterile conditions turned clear colorless AgNO_3_ solutions turbid brown (Fig. 8). These turbid brown solutions like those formed by the plants from non-sterile conditions showed (i) silver NPs specific SPR peak in their absorption spectra (Fig. 8); and (ii) distinct crystalline NPs constituted of Ag (Fig. 9). Also, the PXRD patterns of NPs formed under sterile conditions (Fig. 9), similar to those formed under non-sterile conditions matched with JCPDS No. 89–3722, and 76–1393, indicating presence of Ag^0^ and Ag_2_O. These investigations ruled out the necessity of root associated microorganisms in reduction of Ag^+^ and formation of silver NPs exogenously by root surface of plants.

The root system of plants could effectively reduce ferricyanide to ferrocyanide and DCPIP to colorless DCPIPH_2_ even under strict sterile conditions. Ferricyanide being membrane impermeable, its reduction to ferrocyanide must be occurring exogenously at the root surface, which is also evident from investigations of Rubinstein and co-workers [34]. It is known that ferri to ferro reduction potential (0.430 V) which is significantly higher compared to Ag^+^ to Ag^0^ (0.7991 V) in SHE series [31], [51]. Therefore, root system of plants which have potential to reduce ferricyanide to ferrocynaide can be a powerful system for reduction of Ag^+^ to Ag^0^, at the root surface and form silver NPs.

**Figure 10:**
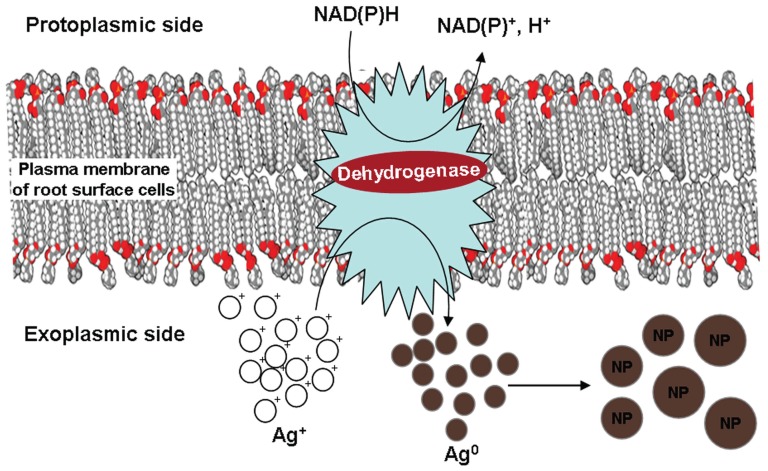
Schematic representation of the mechanism involved in the reduction of Ag^+^ and formation of silver NPs at the root surface of live plants.

It is established that plasma membrane of root surface cells possess transmembrane dehydrogenases/reductases, which play a critical role in root surface mediated reduction processes by drawing electrons from NAD(P)H [33], [34], [52]. This well established fact, makes us believe that the plasma membrane bound dehydrogenase(s)/reductase(s) of root surface cells must have effectively reduced Ag^+^ to Ag^0^ through oxidation of NAD(P)H to NAD(P)^+^. A hypothetical model summarizing the mechanism of reduction of Ag^+^ to Ag^0^ and formation of silver NPs catalyzed by plasma membrane bound dehydrogenase/ reductase is shown in Fig. 10.

### Establishing potential of dehydrogenases to generate silver-NPs

**Figure 11:**
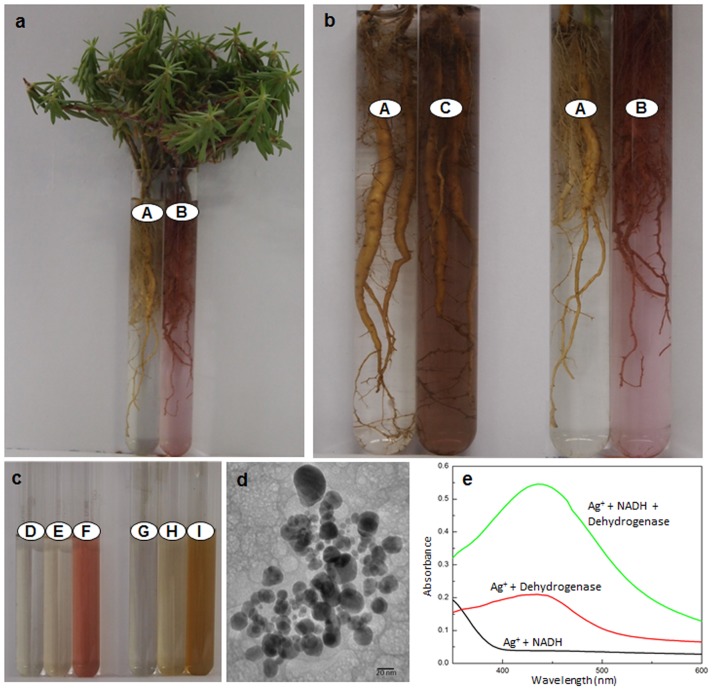
Evidences demonstrating - the presence of dehydrogenases in association with root surface cells (a, b); and the potential of dehydrogenases to reduce triphenyltetrazolium to triphenylformazan and Ag^+^ to Ag^0^ (which in turn generate Ag^0^/Ag_2_O-nanoparticles) (c). (**a**) and (**b**) displays root system of intact plants of *Portulaca grandiflora* incubated in phosphate buffer (pH 7.6) in absence (A) and presence of triphenyltetrazolium chloride (B) or AgNO_3_ (C). Note colour change due to formation of triphenylformazan (B) and silver-nanoparticles (C). (c) depicts tubes with reaction mixture (100 mM phosphate buffer pH 7.6) containing - root enzyme extract with NADH (D); root enzyme extract with triphenyltetrazolium chloride (E); root enzyme extract with triphenyltetrazolium chloride and NADH (F); AgNO_3_ with NADH (G); AgNO_3_ with root enzyme extract (H); and AgNO_3_ with root enzyme extract and NADH (I), 6 h after incubation at 37°C. Please note colour change due to formation of triphenylformazan (F) and silver nanoparticles (I). (**d**) shows silver nanoparticles from tube (I) formed due to dehydrogenase activity in presence of NADH. (**e**) depicts absorption spectra of the reaction mixtures from tubes (G), (H) and (I). Note silver nanoparticle specific absorption peak that intensified due to dehydrogenase activity in presence of NADH.

Roots of intact plants of intact plants of (i) *Triticum aestivum* (raised under sterile conditions); and (ii) *Portulaca grandiflora* reduced clear 2,3,5-triphenyltetrazolium chloride to colloidal pink-purple 1,3,5-triphenylformazan at their surface (Fig. 11) confirming the presence of dehydrogenases in association with root surface cells. Reduction of clear 2,3,5-triphenyltetrazolium chloride to colloidal pink-purple 1,3,5-triphenylformazan is comparable to the conversion of clear AgNO_3_ solution colloidal brown by the root system of intact plants (Fig. 11).

Root enzyme extracts of both *Triticum aestivum* and *Portulaca grandiflora* reduced triphenyltetrazolium to triphenylformazan in presence of NADH, confirming the presence of dehydrogenase activity (Table 2; Fig. 11). Root enzyme extracts of these plant species turned reaction mixture (phosphate buffer, pH 7.6) containing AgNO_3_, colloidal brown within a duration of 3 to 6 h at 37°C, in presence of NADH (Fig. 11). Absorption spectra of these colloidal brown solutions showed presence of silver-NPs specific plasmon resonance peak. Transmission electron microscopic investigations revealed presence of silver-NPs in these solutions. These findings unequivocally demonstrated that dehydrogenases present in the enzyme extract possess potential to reduce Ag^+^ to Ag^0^ and promote formation of silver-NPs.

**Table 2:** Dehydrogenase activities recorded at the surface of roots of intact plants (in vivo) and in root enzyme extracts (in vitro).

S. No.	Plant Species	Dehydrogenase Activity (nmoles g fresh weight^−1^h^−1^)
**In vivo**		
1.	*Portulaca grandiflora*	108.37±11.22
2.	*Triticum aestivum*	898.86±7.67
**In vitro**		
3.	*Portulaca grandiflora*	35.81±2.91
4.	*Triticum aestivum*	572.4±6.18

Root systems of intact plants were incubated in 100 mM phosphate buffer (pH 7.6) containing triphenyltetrazolium chloride at 37°C. Root enzyme extracts were incubated with triphenyltetrazolium chloride and NADH at 37°C. Dehydrogenase activity was determined in terms of nmoles of triphenylformazan formed. Values represent mean ± standard error (n = 5).

### Advantages of employing root system of intact plants for generation of silver NPs

Biological methods for synthesis of nanoparticles are considered to be superior over various physical and chemical methods [18]. Successful generation of silver NPs using variety of microorganisms and plant extracts has been most widely reported. However, due to limitations none of these methods could be used for commercial production of silver NPs. Use of microorganism requires special facilities for their maintenance and safety measures [26]. Amongst microorganisms, even pathogenic microorganisms such as *Klebsiella pneumonia*, *Bacillus subtilis*, *Micrococcus luteus,* S*erratia marcescens*, *Aspergillus* etc. have been employed for generation of silver NPs [21]. However, the serious concern for the use of such pathogenic microorganisms would be likely emergence of new pathogenic strains resistant to silver NPs that are otherwise widely used as antimicrobial agents. Although, synthesis of silver NPs using extracts of various plant materials was reported to be superior, it is difficult to precisely identify the biomolecule(s) amongst the cocktail of molecules in these extracts that are responsible for generation of silver NPs.

Similarly, the potential of live plants to synthesize silver NPs intracellularly cannot be exploited for commercial production due to practical difficulties in their extraction. Moreover, due to the presence of wide variety of biomolecules (having reducing strength) in the cells, intracellularly formed NPs would be of broad size range. Accordingly, Beattie and Haverkamp [31] observed nanoparticles in the range of 2–100 nm in cells of *B*. *juncea* plants exposed to silver salt solutions. In contrast, during present investigations potential of root system of intact plants to exogenously generate silver NPs was realized. Moreover, the silver NPs formed exogenously by root system were in narrow size range. Most importantly, these exogenously generated silver NPs can be harvested with ease.

Amongst chemical methods Lee Miesel protocol for synthesis of silver nanoparticles using sodium citrate continues to remain a standard method, with which newly invented methods are compared [16]. In this popular Lee Meisel method 10 ml of 1% sodium citrate is added to 500 ml of boiling AgNO_3_ solution, and the resultant mixture is boiled for 1 h for synthesis of silver NPs. The major limitation of this method is the production of silver NPs in broad size range i.e. 20–600 nm [15], [16]. In contrast, during present investigations, roots of intact plants of all 16 plant species tested synthesized silver NPs at room temperature within the size range of 5–50 nm.

The potential of root system of intact plants of *V*. *mungo* and *T*. *aestivum* to generate silver NPs was compared with that of sodium citrate at room temperature and under strict sterile conditions. 0.02% sodium citrate (i.e. used for generation of silver NPs in the Lee Meisel method) failed to show any alteration in color of the AgNO_3_ solution or silver NPs specific SPR peak in the absorption spectra even after incubating for duration of 12 h at room temperature. As is evident from Fig. 8 root system of intact plants of *V*. *mungo* and *T*. *aestivum* incubated under ambient and sterile conditions altered the color of clear AgNO_3_ solution colloidal brown within 6 h and the absorption spectra of these colloidal solutions showed silver NPs specific SPR peak. Although, sodium citrate when used at a concentration 50 times higher than that used in Lee Meisel method, turned clear colorless AgNO_3_ solutions grayish colloidal, the intensity of color and silver NPs specific SPR peak in the absorption spectra of these colloidal solutions was significantly less than that recorded when root system of intact plants were used, under ambient conditions (Fig. 8). The dynamic light scattering (DLS) studies showed that the mean particle size of silver NPs synthesized using 1 mM AgNO_3_, by the root system of intact plants of *V*. *mungo* and *T*. *aestivum* was ∼20 nm, while those synthesized in presence of 1% sodium citrate was ∼33 nm under ambient conditions. These findings, clearly demonstrate that root system of intact plants can be employed for rapid synthesis of silver NPs under ambient conditions. To the best of our knowledge, the novel method of using root system of intact plants for bulk synthesis of silver NPs under ambient conditions would be most green, simple and economically viable.

## Conclusions

The findings presented in this manuscript demonstrated for the first time that root system of intact plants possess immense potential to reduce Ag^+^ and form Ag^0^/Ag_2_O NPs. Using 16 plant species from 11 diverse families, it has been demonstrated that the root system of all angiosperms possess reducing strength that can be aptly used for reduction of metal ions like Ag^+^. Root system of plants could reduce Ag^+^ and generate Ag^0^/Ag_2_O NPs even under strict sterile conditions, establishing that root system alone possess immense reducing strength. Efficacy of root system of these plant species to reduce membrane impermeable ferricyanide to ferrocyanide and triphenyltetrazolium to triphenylformazan established that plasma membrane bound dehydrogenases/reductases are responsible for reduction of Ag^+^ at surface, and exogenous generation of Ag^0^/Ag_2_O NPs. Dehydrogenases in root enzyme extract possessed potential to reduce Ag^+^ to Ag^0^ and generate Ag^0^/Ag_2_O NPs in the presence of NADH. As the plasma membrane bound dehydrogenases seems to be a single major component for reduction of Ag^+^ the NPs generated were relatively more or less spherical and in narrow size range. The findings presented in this communication also established that synthesis of Ag^0^/Ag_2_O NPs by root system of intact plants is superior over all the biological methods reported so far. To the best of our knowledge, this novel method for generation of silver NPs is most green, simple, and economically viable.
